# Supplementary material for: The Kinase Specificity of Protein Kinase Inhibitor Peptide
Source: Front Pharmacol. 2021 Jan 29;12:632815. doi: 10.3389/fphar.2021.632815 (PMC7878667; doi:10.3389/fphar.2021.632815)
Supplement: Supplementary file 1 [file DataSheet1.pdf]

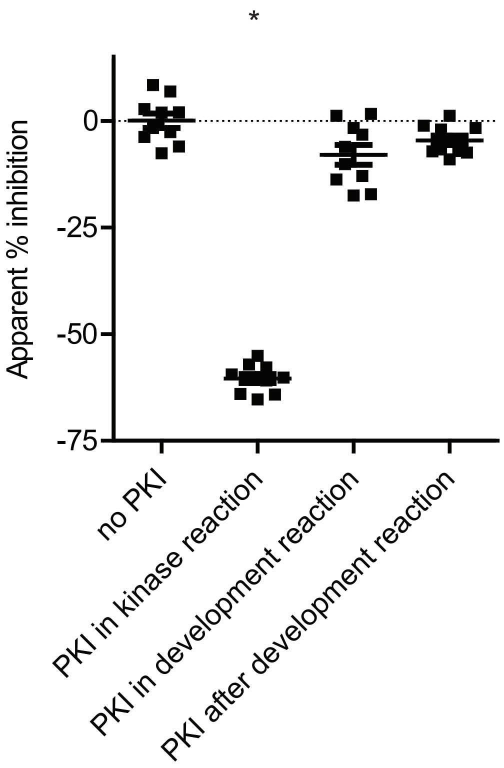

Supplementary Figure 1. PKI (6-22) amide inhibits kinase reaction without significant effect on the protease/development reaction or fluorescence of the substrate. Apparent % inhibition, based on FRET ratio, of adding PKI in the kinase reaction of PRKCE (PKC epsilon), the protease development reaction, or after development reaction. *: $p<0.05$ vs. no PKI, $p<0.05$ vs. PKI in development reaction, $p<0.05$ vs. after development reaction (Kruskal-Wallis followed by Dunn's Multiple Comparison Test). The graph shows individual data points (squares) as well as median with interquartile range (lines).
